# *Achromobacter* spp. Surgical Site Infections: A Systematic Review of Case Reports and Case Series

**DOI:** 10.3390/microorganisms9122471

**Published:** 2021-11-30

**Authors:** Eve Ronin, Christian Derancourt, André Cabié, Karine Marion-Sanchez

**Affiliations:** 1 Department of Hospital Hygiene, CHU Martinique, F-97200 Fort-de-France, Martinique; roninevealizee@gmail.com; 2 Department of Internal Medicine, CH Gap, F-05007 Gap, France; christian.derancourt@chicas-gap.fr; 3 Department of Infectious Diseases, CHU Martinique, F-97200 Fort-de-France, Martinique; andre.cabie@chu-martinique.fr; 4 Unité Mixte de Recherche 1058 : Pathogenesis and Control of Chronic and Emerging Infections, 34000 Montpellier, France

**Keywords:** *Achromobacter* spp., surgical site infection, immunocompetent, review

## Abstract

*Achromobacter* species are isolated from rare but severe healthcare-associated infections, including surgical site infections. They are considered to preferentially infect immunocompromised patients but so far with limited evidence. We conducted a systematic review on *Achromobacter* spp. surgical site infections (SSIs) to determine if such infections were indeed more commonly associated with immunocompromised patients. The secondary objective was to describe the characteristics of infected patients. Eligible articles had to be published before 30 September 2020 and to report *Achromobacter* spp. SSIs across all surgical specialties excluding ophthalmology. Analyses were performed on individual data without meta-analysis. Cases were divided into 2 subgroups: one group which had either prosthesis or implant and the other group which did not. A first selection led to a review of 94 articles, of which 37 were analyzed. All were case reports or case series and corresponded to 49 infected patients. Most of the patients were under 65 years of age and had undergone a heart or digestive surgery followed by deep infection with no co-infecting pathogens. Nine out of the 49 cases were immunocompromised, with similar distribution between the two subgroups (16.6% and 20%, respectively). This review suggests that *Achromobacter* spp. SSIs do not preferentially target immunocompromised patients.

## 1. Introduction

Surgical Site Infections (SSIs) are a major cause of increased morbidity and mortality, prolonged hospital length of stay, hospital readmissions, and increased health care costs in surgical patients [1]. According to the results of the latest Nosocomial Infections National Prevalence Survey (NINPS), SSIs accounted for 20% of all the Healthcare-Associated Infections (HAIs) in 2017 in the metropolitan and the overseas regions of France and represented the second most common HAI after the urinary tract infections [2]. The bacteria commonly isolated in SSIs are *Staphylococcus aureus* and *Enterobacteriaceae*, but environmental bacteria such as *Pseudomonas aeruginosa* account for a significant proportion of the agents involved in such infections [3].

*Achromobacter* species, especially *A.xylosoxidans*, have also been isolated from rare but severe HAIs including SSIs [4,5,6,7,8,9]. Initially described as low-virulence and sporadic contaminants, these bacteria have been emerging in health care facilities for the past few decades. Knowledge about these microorganisms is continuously increasing [10] with some virulence factors identified [11] and some case reports describing severe infections with fatal outcomes [11,12,13,14,15]. Most authors present *Achromobacter* spp. as an opportunistic pathogen that preferentially infects immunocompromised patients, but so far with insufficient amounts of evidence [6,16,17,18,19,20,21,22].

We hypothesize that patients with *Achromobacter* SSIs are not predominantly immunocompromised. A preliminary search in MEDLINE via PubMed database and in PROSPERO registry showed the lack of systematic review on *Achromobacter* spp. surgical site infections, neither published, nor in development. To explore our hypothesis, we conducted a systematic review on *Achromobacter* spp. SSIs across all surgical specialties. Our primary objective was to determine if *Achromobacter* spp. SSIs preferentially target immunocompromised patients. The secondary objective was to describe the main demographic and clinical characteristics of infected patients.

## 2. Materials and Methods

This systematic review was conducted following the PRISMA (Preferred Reporting Items for Systematic Reviews and Meta-Analyses) recommendation.

The research question was: do *Achromobacter* spp. mediated SSIs preferentially target immunocompromised patients?

### 2.1. Search Strategy

A comprehensive literature search was performed in the following databases, registries and search engines: MEDLINE via PubMed, Cochrane Library, Clinicaltrials.gov, GoogleScholar.com, Theses.fr. The search was completed by the analysis of “similar situations” on e-sin.santepubliquefrance.fr, the French reporting platform for healthcare-associated infections, and by the examination of the discussion board of cpias-ile-de-france.fr, a French website for the prevention of healthcare-associated infections. The search covered all publications up to and, including 30 September 2020, with no start date specified. Search terms were defined by two independent researchers and adapted to each repository by combining vocabulary, relevant MeSH terms, and keywords such as “Achromobacter”, “Alcaligenes xylosoxidans”, “surgery”, “surgical site infection”, “wound infection“, “nosocomial infection”, ” healthcare-associated infection”, “bacteraemia”, “endocarditis”, “meningitis”, “bacteraemia”, “ventriculitis”, “mediastinitis”,” abscess”, “peritonitis”, “burns”. When available, filters were applied to select studies in English and French languages and include those involving human subjects only. Additional studies were identified from the references provided by retrieved studies. The detailed search strategy adapted to each database or repository is provided as Supplementary File S1.

### 2.2. Eligibility Criteria

A study was eligible for inclusion if it described at least one case of either deep or superficial *Achromobacter* spp. SSI in patients across all ages and included documentation of clinical and microbiological criteria, regardless of the length of the time interval between surgery and infection. The eligibility included the SSIs occurring either in the surgical wounds, spaces, or organs, and/or on prosthetic materials or implants which were placed during surgery. All types of surgery were considered except for ophthalmic surgery.

*Achromobacter* spp. infection, either superficial or deep, was considered an SSI if it met the clinical and microbiological criteria specified by the Center for Disease Control [23]. With regard to the timing of onset, the French definition, which is more precise for this criterion, was preferred. An SSI was then defined as an HAI if it occurred within 30 days post-surgical procedure that did not involve an implant or prosthesis, and within one year when either implant or prosthesis was involved in the surgery process. Beyond this period, an SSI was considered a community-acquired infection unless authors established a clear link between surgery and infection [24].

All included articles had to provide sufficient information on the patients’ immune status. Unless the “immunocompetent” status was clearly announced, patients with at least one of the following criteria described in the NINPS protocol, i.e., solid tumor or hematological malignancy, organ transplantation, radiotherapy, chemotherapy, immunosuppressive therapy, high-dose prednisolone (>5 mg/kg/day) or prolonged (>30 days) use of corticosteroids or Human Immunodeficiency Virus infection with <500 CD4^+^ cells/mm^3^ [25] were defined as immunocompromised. In contrast, co-morbidity factors (i.e., diabetes mellitus, obesity), which are known to predispose individuals to various infections [26], were not taken into account.

Articles dealing with fundamental science and general guidelines were excluded as well as those that described the surgical site colonization only.

### 2.3. Study Selection

Study selection was performed by two independent researchers using the Microsoft Excel software. Titles of potentially eligible studies were integrated into an Excel spreadsheet. By applying a color code and alphabetic classification, duplicates were eliminated and selection on titles and abstracts was performed online. The two independent researchers examined the selected full-text materials to ensure the accuracy and the eligibility of the criteria. In case of a disagreement between the two researchers, an opinion from a third researcher was requested. The case was discussed between the three researchers until a consensus was reached.

For recent studies (publication year > 2010), when the patient’s immune status or individual data were not provided, the authors of the studies were contacted for the missing data.

### 2.4. Data Collection Process and Items

Independent double extraction of data from eligible, full-text articles was performed by two researchers, each following the same extraction form. For each article, the following data were recorded: name of the first author, the year of publication, demographics (age and sex), immune status, surgical data (surgical specialty, date of surgery, surgical site), infection data (the time interval between surgery and diagnosis, depth, identification of *Achromobacter* spp., clinical criteria for diagnosis, associated microorganisms), treatments and outcome. The time period between surgery and diagnosis was defined as an interval between the surgery and the date of the first positive sample with *Achromobacter* spp..

Cases with some missing data were accepted as long as the information on the patient’s immune status was available.

### 2.5. Data Analysis

The analysis was carried out on the individual data file using the Microsoft Excel software. Categorical data were expressed as percentages (numbers). Quantitative data were expressed as a median [min-max] and means +/− standard deviation.

## 3. Results

### 3.1. Study Selection

**Figure 1:**
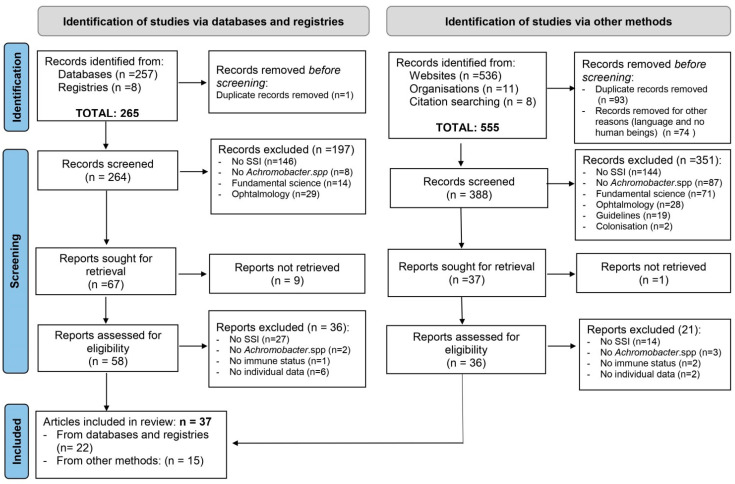
PRISMA flow diagram.

PRISMA 2020 flow diagram (Figure 1) describes the different stages of study selection. A total of 820 records were identified by our literature search. Among these records, 94 were duplicates and therefore, removed. After examining the titles and abstracts, 548 records were excluded according to our eligibility criteria. Ninety four full-text materials were examined in their entirety. The authors from 5 cases were contacted for missing information but these authors were unresponsive. Finally, 37 articles were included in the analysis [4,6,7,8,12,13,14,16,17,18,19,20,21,22,27,28,29,30,31,32,33,34,35,36,37,38,39,40,41,42,43,44,45,46,47,48,49].

### 3.2. Study Characteristics

The extensive literature search led to the inclusion of case reports or case series only. These cases were published between 1960 and 2020. Thirty articles described isolated case reports; seven articles described clustered cases of infections i.e., liver abscesses, meningitis, skin and soft tissue infections, bacteremia, pediatric infections, and infections associated with medical devices [4,6,13,20,29,30,31]. SSIs were extracted from these cluster cases. Finally, a total of 49 different cases were included in the study.

According to Köppen’s climatic classification [50], 36 cases (73%) were from tropical, humid subtropical, or hot summer Mediterranean climates which are characterized by high heat and humidity.

The main characteristics of the final selections are shown in Table 1.

### 3.3. Descriptive Analysis of Included Cases

Since our final selection was limited to cases and cluster cases with significant methodological heterogeneity, it was not possible to carry out a meta-analysis. Therefore, results will be presented in narrative form. For descriptive analysis, two subgroups were defined according to the presence (subgroup 1, *n* = 24) or absence (subgroup 2, *n* = 25) of either prosthesis or implant (Table 1). Because of the small sample size, no statistical comparisons were performed on these two subgroups.

#### 3.3.1. Demographics

The median and mean ages were similar between subgroup 1 (45.5 years [min: 0.16, max: 86]; mean = 44 +/− 23.4) and subgroup 2 (47 years [min: 4, max: 74]; mean = 45.3 +/− 19); Forty of the 49 patients included (81.6%) were under 65 years of age. The M/F sex ratios were 13/11 in subgroup 1 and 16/9 in subgroup 2 (Table 1).

#### 3.3.2. The Patients’ Immune Status

Only 9 of the 49 recorded cases (18.3%) were immunocompromised, with similar distribution between the two subgroups (16.6% and 20%, respectively) (Table 1). Five patients had solid tumors, 1 patient had Hodgkin’s lymphoma, 1 patient had rheumatoid arthritis, 1 patient had undergone splenectomy and 1 patient was a burn victim. Therefore, the remaining 81.7% of the cases were immunocompetent patients.

#### 3.3.3. Surgical Data and Infections

Surgical data are provided in Table 2. The main pathologies leading to surgery were heart diseases (30.6%, *n* = 15), digestive pathologies (22.4%, *n* = 11) and neurological impairments (14.3%, *n* = 7). The 80% of total cases presented were deep infections.

In subgroup 1, the most represented surgery was cardiac surgery (54.1%, *n* = 13); 95.9% (*n* = 23) of SSIs were deep infections and occurred mainly after thoracotomy (9 endocarditis, 3 mediastinitis, and 2 abscesses). Only one superficial wound infection was described after total knee arthroplasty (case no 10). The time of onset was recorded for 21 out of 24 SSIs and 16 (76.2%) met the criteria for nosocomial infection. In other words, only 5 infections occurred more than one year after surgery.

In subgroup 2, the most represented surgery was digestive surgery (32%, *n* = 8); 68% (*n* = 17) of SSIs were deep infections and occurred mainly after digestive surgery (1 peritonitis and 7 abscesses). The time of onset was described for 19 out of 25 SSIs and 9 (47.3%) occurred within one month of surgery, thus meeting the criteria for a nosocomial infection.

#### 3.3.4. Isolated Strains

The species responsible for the infection was identified as *Achromobacter xylosoxidans* in 84.4% of the included cases (*n* = 38) (Table 2). For the 11 other cases, identification led to *Achromobacter* spp. (*n* = 9), *Achromobacter Group B* (*n* = 1) and *Achromobacter Group Vd Biovar* (*n* = 1). The method of strain identification was indicated in 51% of cases (*n* = 25) only. The most commonly used method was culture and biochemical tests (*n* = 17). Only 4 studies involving a total of 8 cases including our previous case report utilized molecular detection methods. Of these studies, only one case described the detection and identification via nrdA gene sequencing [12].

Most cases in both subgroup 1 (91.6%, *n* = 22) and 2 (72%, *n* = 18) were not co-infected. The main co-infecting microorganisms detected in both subgroups belonged to the family of Enterobacteriaceae.

#### 3.3.5. Treatments and Outcomes

Detailed information about antibiotic therapy was available for 87.7% of cases (*n* = 43) (Table 2). For 27 of these 43 cases (62.7%), an empirical antibiotic therapy was initiated. Antibiotic sensitivity of isolated strains was provided for 41 cases out of 49. Based on available data, a total of 30 out of 41 cases had an antibiotic therapy adapted to strain sensitivity (73.2%). Carbapenems (Imipenem, Meropenem and Ertapenem) were the most prescribed drugs (*n* = 18), followed by Cotrimoxazole^®^ (*n* = 8), Quinolones (*n* = 6) and Tazocillin^®^ (*n* = 7).

Outcome was indicated for 49 cases. Ten patients died. Global mortality rate was 20.4% with 25% (*n* = 6) for subgroup 1 and 16% (*n* = 4) for subgroup 2. Only 2 out of 9 immunocompromised patients died (22.2%).

In subgroup 1, 12 patients (50%) underwent surgical revision involving the removal of prosthetic material and all 12 patients survived. Conversely, 50% of the remaining patients (*n* = 6) who did not undergo such removal died. In this subgroup, 19 out of 24 (79.1%) patients received an antibiotic therapy adapted to sensitivity but 5 of these treated patients died with implanted materials still in place.

#### 3.3.6. Origin of Infection

A possible origin of infection was proposed for 14 cases. The most suspected sources were contaminated waters (Table 2). Three cases were extracted from an outbreak associated with 6 *Achromobacter* spp. cerebral ventriculitis in the neurosurgical ward and the source was confirmed as a contaminated aqueous chlorhexidine solution [31]. For other suspected environmental sources, no analysis was performed.

## 4. Discussion

This review supports our hypothesis stating that *Achromobacter* spp. SSIs do not preferentially target immunocompromised patients. To our knowledge, this is the first systematic review of post-surgical infections associated with this bacterium. It provides an overview of reported cases over a period of 60 years showing that the majority of published cases on *Achromobacter* spp. SSIs involve individuals under the age of 65 who mainly develop deep infections after a heart surgery involving either prosthesis or implant or after a digestive surgery that did not involve prosthetic materials. As we have previously suggested [4], these infections seem to preferentially affect patients living in warm and humid environments. However, this observation needs to be confirmed by further studies.

The very low rate of immunocompromised patients is consistent with our hypothesis and also with a recent publication demonstrating that both immunosuppressed and immunocompetent populations can be potential targets for *Achromobacter* spp. healthcare-associated infections [51]. These findings also strengthen our previous finding showing a higher rate (56.1%) of such infections occurring in immunocompetent patients [4]. In addition, a thorough examination of the articles revealed that while the link between immunosuppression and *Achromobacter* spp. infections is initially proposed by almost all the studies either in the introduction or in the discussion section, the data presented in these studies suggest otherwise [6,16,17,18,19,20,21,22]. In our study, we consider actual immunosuppression (defined in the NINPS protocol), not the weakening of the immune system due to chronic illness, implanted devices, even hospitalization or surgery itself; such criteria favor any type of infection related to any microorganism, especially opportunistic ones [26]. Studying such a relationship would not have been of interest since it is already known.

The majority of the cases included are said to be infected with *Achromobacter xylosoxidans*. However, the validity of such a level of identification remains questionable. To our knowledge, the nrdA gene sequencing is the only method that allows identification of *Achromobacter* isolates down to species level [52] and so far, there is only one study (authored by this group) that utilized this method of detection [12]. On another hand, it is important to note that many of the studies included in this review predate the time when molecular and genetic methods of bacterial identifications were available.

Lack of a greater number of series, heterogeneity of available evidence base, and publication bias are potential limitations of this study and these limitations may preclude us from reaching definitive conclusions. Moreover, we did not conduct the assessment of the methodological quality of each included finding, which may result in confounding, selection, and information bias.

The lower rate of immunocompromised patients could be interpreted as being related to the extreme scarcity of *Achromobacter* spp. infections among immunocompetent patients, leading to more frequent publications of these rare cases. But on the other hand, *Achromobacter* spp. SSIs are sufficiently rare to be reported regardless of the immune status of patients.

Similarly, our study shows higher death rates among the reported cases (26.1% for subgroup 1 and 14.3% for subgroup 2) than those associated with other SSI-causing microorganisms (from 2.5 to 6% depending on the study) [53]. Surprisingly, the death rate was not higher among immunocompromised patients. The high mortality rates may be related to publication bias with a majority of severe cases being published at the expense of mild cases. Fifty percent of these deaths can be explained by the pathology itself, i.e., severe cardiac damage requiring implants or artificial valves and leading to mediastinitis or endocarditis. On the other hand, deaths following cholecystectomy in patients without other comorbidity are more questionable. Retrospective studies on *Achromobacter* spp. HAIs report death rates of 22% [54] and 15% populations [9] which are similar rates to those observed in the present study. The hypothesis of high mortality rates being associated specifically with *Achromobacter* spp. itself, therefore, cannot be fully ruled out.

Some of the delays between the surgery and the infection were extremely lengthy. The lengthiest was an intra-cardiac abscess occurring 29 years after surgery [35]. Either a greater number of extremely late infections have been published because of their impressive character, or there is a specific cause of delayed infection. Perhaps *Achromobacter* spp. deep SSIs develop very slowly over time while remaining in transient dormancy and most likely in biofilm communities; delayed diagnosis may also be responsible for the prolonged onset because of misidentification or mild, nonspecific clinical signs at initial diagnosis [14,17,29]; finally, delayed *Achromobacter* infections are more likely due to delayed acquisition in the hospital environment.

Prevention and treatment of SSI itself present significant challenges and it can be further exacerbated by the intrinsic nature of bacteria causing the infection such as seen in many SSI cases associated with *Achromobacter* spp.. First, this bacterium was shown to acquire resistance to the antiseptic solutions used for skin preparation. Three cases were concerned by the growth of this microorganism inside aqueous chlorhexidine solutions [31]. Second, *Achromobacter* spp. is able to grow in sterile distilled water [55]. We previously isolated this organism from a patient who was treated with V.A.C. therapy associated with sterile water irrigation. It is plausible that this treatment may have supported the growth of this microorganism [12] and therefore worsened the existing infection. Third, *Achromoabcter spp* is known to form biofilms [55,56] which favor the transfer of resistance genes as well as the development of bacterial tolerance to antimicrobial agents. Biofilm growth may explain a higher rate of deep infections observed in patients with prostheses or implants and subsequent challenges these patients encountered during antibiotic therapy. Although many of the patients seem to have benefited from adapted antibiotic therapy, the removal of implanted materials appears to be the only treatment that ensured survival. In accordance with the literature, Carbapenem were the most prescribed antibiotics regardless of the presence or absence of foreign materials in patients’ bodies. Unfortunately, frequent and widespread usage of these molecules has favored the increase in acquired resistance [57]. New therapeutics against carbapenem-resistant Gram-negative non-fermenters are emerging; Cefiderocol, a new generation cephalosporin, has recently been shown efficacious in the treatment of *Achromobacter* post-operative osteomyelitis [57].

## 5. Conclusions

This review suggests that *Achromobacter* spp. surgical site infections can affect patients independent of their immune status. Patients living in hot and humid climates may be at a greater risk of acquiring *Achromobacter* spp. SSIs but effective preventive measures can be proposed only when the source of such infections are clearly identified. The association between *Achromobacter* spp. infections and climate needs to be thoroughly investigated. Future studies including a greater number of case series and similar reviews extended to all types of healthcare-associated infections caused by *Achromobacter* spp. are recommended.

## Figures and Tables

**Table 1:** Case characteristics and surgical procedures.

Scheme 1. Surgical Procedures Including Prosthesis or Implant								
Case Characteristics							Surgical Procedures	
Case no.	Author year (ref)	Country State/city Climate *	Age (Y)	Sex	Main Disease and Associated Pathologies	Immune Status	Type of Surgery	Description
1	Derber 2011 [6]	USA Virginie Cfa	54	F	Tetralogy of Fallot, Blalock-Taussig shunt as a child, total repair as a teenager	Comp	Heart	Bovine pulmonary valve replacement
2	Gelfand 2014 [28]	USA Tennessee Dfa	29	F	Spina Bifida and placement of a ventriculo-peritoneal shunt in early childhood.	Comp	Neurological	Replacement of VP shunt
3	Gupta 2012 [17]	India Lucknow Csa	65	M	Small paraumbilical hernia	Comp	Digestive	Open mesh repair
4	Van Hal 2008 [18]	Australia Sydney Cfa	37	M	Native valve endocarditis, intravenous drug user	Comp	Heart	Aortic valve replacement
5	Olson 1981 [14]	USA California Csa	35	M	Dissection of the aortic root, aortic stenosis, and insufficiency	Comp	Heart	Aortic resection and Daflon tube graft insertion
6	Santaeufemia 2014 [30]	Spain Madrid Csa	0.16	F	Congenital hydrocephalus	Comp	Neurological	Reducer cranioplasty and VP shunt
7	Sawant 2013 [19]	USA California Csa	62	F	Chronic heart failure, chronic obstructive pulmonary disease, chronic kidney disease	Comp	Heart	Bioprosthetic aortic valve replacement and placement of pace-maker
8	Shigeta 1978 [31]	Japan Fukushima Cfa	9	F	Arachnoid cyst	Comp	Neurological	Arachnoid cyst and VP shunt
9	Shigeta 1978 [31]	Japan Fukushima Cfa	8	M	Thalamic tumor	Supp	Neurological	VP shunt
10	Tena 2014 [32]	Spain Guadalajara Csa	57	F	No underlying disease	Comp	Orthopedic	Total knee arthroplasty
11	Ahn 2004 [21]	Korea Iksan Dfa	35	M	Chronic heart failure	Comp	Heart	Pacemaker placement
12	Padmaja 2013 [8]	India Hyderabad Aw	17	M	Congenital aortic stenosis	Comp	Heart	Prosthetic aortic valve replacement
13	Rafael 2014 [35]	USA Ohio Dfa	50	F	Splenomegaly, pancytopenia, ventricular septal defect	Supp	Heart	Ventricular septal defect repair
14	Linde 1960 [44]	USA California Csa	20	M	Tetralogy of Fallot	Comp	Heart	Definitive repair using Ivalon patch
15	Tokuyasu 2012 [36]	Japan Tottori Cfa	86	F	Chronic heart disease	Comp	Heart	Prosthetic aortic valve replacement
16	Taylor 1991 [37]	USA Missouri Dfa	53	F	Rheumatoid arthritis	Supp	Orthopedic	Prosthetic knee replacement
17	Bhattarai 2016 [22]	USA Illinois Dfa	37	F	Chronic heart disease, intravenous drug user	Comp	Heart	Mitral valve replacement
18	Marion- Sanchez 2018 [12]	Martinique Fort-de-France AM	81	M	Chronic heart disease	Comp	Heart	Mitral valve remplacement
19	MarionSanchez 2019 [4]	Martinique Fort-de-France AM	58	M	Digestive ulcer	Comp	Thoracic	Esophageal stent placement
20	MarionSanchez 2019 [4]	Martinique Fort-de-France AM	41	M	Biliary stenosis	Comp	Digestive	PEG tube placement
21	Lofgren 1981 [42]	USA Minneapolis Dfa	77	F	Rheumatic aortic stenosis	Comp	Heart	Prosthetic aortic valve replacement
22	Tripathi 2020 [41]	USA Lexington Cfa	65	M	Distal common bile duct stone, chronic alcoholism, tonsillar adenocarcinoma	Supp	Digestive	PEG tube placement
23	McKinley 1989 [45]	UK Aylesbury Cfb	28	M	Aortic valve regurgitation	Comp	Heart	Aortic valve replacement
24	Lee 2014 [46]	Korea Seoul Dwa	52	M	Osteoarthritis	Comp	Orthopedic	Total knee arthroplasty
**Subgroup 2—Surgical Procedures without Prosthesis or Implant**								
**Case Characteristics**							**Surgical Procedures**	
**Case** **no.**	**Author Year (Ref)**	Country State/City Climate *	**Age (Y)**	**Sex**	**Main Disease and** **Associated Pathologies**	**Immune Status**	**Type of Surgery**	**Description**
25	Ferroir 1988 [27]	France Paris Cfb	54	F	Pneumocephalus, Hodgkin disease	Supp	Thoracic	Lobectomy
26	Holmes 1977 [29]	UK London Cfb	38	F	Breast Carcinoma, steroid, and hormonal therapy, chemo and radiotherapy.	Supp	Gynecological	Mastectomy
27	Ozer 2009 [16]	Turkey Hatay Csa	38	M	Traffic accident, central facial paralysis	Comp	Neurological	Removal of arachnoid cyst by craniotomy
28	Santaeufemia 2014 [30]	Spain Madrid Csa	13	M	Trafic accident, open fracture of the proximal left tibia and fibula	Comp	Orthopedic	Osteosynthesis
29	Shigeta 1978 [31]	Japan, Fukushima Cfa	49	M	Meningioma	Supp	Neurological	Meningioma resection by craniotomy
30	Tena 2014 [32]	Spain Guadalajara Csa	47	F	Mandibular abscess	Comp	Stomatological	Submandibular abscess debridement
31	Tena 2014 [32]	Spain Guadalajara Csa	18	H	Pilonidal cyst	Comp	Digestive	Cyst removal
32	Tsay 2005 [20]	Taïwan Changhua Cfa	46	M	Empyema	Comp	Thoracic	Decortication of empyema
33	D’Amato 1988 [33]	USA New York Dfb	14	M	Totally transected spinal cord after gunshot to the chest	Comp	Thoracic	Ligation of bleeding vessels and repair of lung laceration
34	Asano 2005 [13]	Japan Kumamoto Cfa	52	F	Cholecystolithiasis	Comp	Digestive	Cholecystectomy
35	Asano 2005 [13]	Japan Kumamoto Cfa	57	M	Cholecystolithiasis	Comp	Digestive	Cholecystectomy
36	Asano 2005 [13]	Japan Kumamoto Cfa	52	M	Cholecystolithiasis	Comp	Digestive	Cholecystectomy
37	Vinod 2013 [7]	India Kerala AM	51	M	Simple renal cyst	Comp	Neurological	Laparoscopic deroofing of a simple renal cyst
38	Teng 2009 [34]	Taïwan Taipei Cfa	27	M	Cholecystolithiasis	Comp	Digestive	Cholecystectomy
39	CPIAS 2003 [38]	France Paris Cfb	36	M	Achilles tendon rupture	Comp	Orthopedic	Achilles tendon
40	Rotter 2020 [39]	USA Minnesota Dfb	70	M	Mucocele	Comp	Neurological	Resection of left frontal sinus and zygomatico-maxillary mucocele
41	Sari 2018 [40]	Turkey Yozgat Dsb	56	M	Kidney stone	Comp	Nephro- logical	Intrarenal surgery for kidney stone
42	MarionSanchez 2019 [4]	Martinique Fort-de-France AM	74	M	History of colorectal Adenocarcinoma, Peritonitis	Supp	Digestive	Exploratory laparotomy
43	MarionSanchez 2019 [4]	Martinique Fort-de-France AM	63	F	Chronic heart disease	Comp	Heart	Double coronary bypass
44	MarionSanchez 2019 [4]	Martinique Fort-de-France AM	65	F	Arteritis	Comp	Vascular	Femorofemoral bypass
45	Linde 1960 [44]	USA California Csa	4	M	Defect of interventricular septum	Comp	Heart	Direct closure
46	Zhi Yang 2014 [43]	Singapore Af	46	F	Extensive thermal burns (41.5% of the total body)	Supp	Reconstructive	Burn excision and staged, free and cadaveric skin grafting
47	Revati 2019 [47]	India Kerala AM	40	F	Cholelithiasis	Comp	Digestive	Cholecystectomy
48	Appelbaum 1980 [48]	USA Pennsylvania Cfa	75	M	Cholecystitis	Comp	Digestive	Laparotomy
49	Demirel 2015 [49]	Turkey Istanbul Csa	59	F	Bladder tumor	Supp	Urological	Transurethral tumorectomy

* According to Köppen climate classification. Y: years; Comp: immunocompetent; Supp: immunosuppressed; M: male; F: female; VP: ventriculo-peritoneal.

**Table 2:** Infection topographies, treatments, and outcomes.

Subgroup 1—Surgical Procedures Including Prosthesis or Implant						
	Infections Topographies				Treatments and Outcomes	
Case no.	Infection, Depth	Delay (Days)	Identified Species/Associated Bacteria	Suspected Origin of Infection	Treatments	Outcomes
1	Endocarditis, deep	120	*Ax subsp. denitrificans*/ No	Ukn	Piperacillin/Tazobactam, Imipenem/Cilastatin/ Levofloxacin	Recov
2	Ventriculitis, deep	30	*Ax*/No	Ukn	Doripenem	Recov
3	Abscess, deep	Ukn	*Ax*/No	Mesh	Ceftriaxone, Levofloxacin	Recov
4	Endocarditis deep	180	*Ax*/No	Duckpond water used to solubilize drugs for injection	Meropenem	Recov
5	Mediastinitis, deep	180	*Ax*/No	Ukn	Carbenicillin/ Cotrimoxazole/Rifampin, Moxalactam/Rifampin, Azlocillin/Rifampin	Death
6	Ventriculitis, deep	20	*Ax*/No	Ukn	Ceftazidime/Meropenem	Recov
7	Endocarditis, deep	90	*Ax*/No	Exposing leg ulcers to water outdoors	Piperacillin/Tazobactam, Meropenem/ Cotrimoxazole, Meropenem/Rifampin/Amikacin	Recov
8	Ventriculitis, deep	12	*Ax*/No	Aqueous Chlorhexidine solution diluted with non-sterile tap water.	Chloramphenicol	Recov
9	Ventriculitis, deep	30	*Ax/Serratia marcescens*	Aqueous Chlorhexidine solution diluted with non-sterile tap water.	Chloramphenicol	Recov
10	Wound infection, superficial	Ukn	*Ax*/No	Ukn	Ukn	Recov
11	Endocarditis, deep	2920	*Ax*/No	Scaling and root planning at local dental clinic	Ceftazidime/Piperacillin	Recov
12	Endocarditis, deep	150	*Ax subsp. denitrificans*/No	Ukn	Meropenem/ Levofloxacin, Meropenem/Cotrimoxazole	Recov
13	Intracardiac abscess, deep	10,585	*Ax*/No	Ukn	Piperacillin/ Tazobactam/Cotrimoxazole	Recov
14	Endocarditis, deep	3	*Achromobacter* spp./No	Contamination of extracorporal heart pump	Chloramphenicol/Sulfonamides	Death
15	Endocarditis, deep	1825	*Ax*/No	Ukn	Meropenem	Death
16	Abscess, deep	Ukn	*Ax*/No	Ukn	Ceftazidime, Imipenem/Cotrimoxazole	Recov
17	Endocarditis, deep	Ukn	*Ax*/No	Cocaïne mixed with stored tap water	Meropenem	Recov
18	Mediastinitis, deep	20	Ax/*Staphylococcus aureus*	Water leaks in the ceiling in intensive care unit	Piperacillin/Tazobactam, Meropenem/Vancomycin, Ceftazidime	Death
19	Mediastinitis, deep	10	*Achromobacter* spp./No	Ukn	Meropenem/Vancomycin	Recov
20	Abscess, deep	90	*Achromobacter spp/*No	Ukn	Tazobactam	Recov
21	Endocarditis, deep	120	*Ax*/No	Ukn	Cotrimoxazole/Moxalactam	Death
22	Peritonitis, deep	16	*Achromobacter* spp./No	Ukn	Piperacillin/Tazobactam, Meropenem/Vancomycin	Death
23	Endocarditis, deep	135	*Achromobacter Group B*/No	Ukn	Cefuroxime/Gentamycin	Recov
24	Prosthetic infection deep	395	*Ax/*No	Ukn	Cefazolin Ciprofloxacin Imipenem	Recov
**Subgroup 2—Surgical Procedures without Prosthesis or Implant**						
	**Infections Topographies**				**Treatments and Outcomes**	
**Case** **no.**	**Infection, Depth**	**Delay (Days)**	**Identified Species/Associated Bacteria**	**Suspected Origin of Infection**	**Treatments**	**Outcomes**
25	Meningitis, deep	15	*Ax*/No	Aerosol	Metronidazole/Imipenem	Recov
26	Wound infection, superficial	270	*Ax*/No	Ukn	Ukn	Ukn
27	Meningitis, deep	3	*Ax*/No	Ukn	Meropenem	Recov
28	Wound infection, superficial	11	*Ax*/No	Ukn	Imipenem, Vancomycin	Recov
29	Ventriculitis, deep	6	*Ax/*No	Aqueous Chlorhexidine solution diluted with non-sterile tap water.	Ukn	Recov
30	Cervical abscess, superficial	Ukn	*Ax*/*Candida albicans*	Ukn	Ciprofloxacin	Recov
31	Gluteal abscess, superficial	Ukn	*Ax*/No	Ukn	Cotrimoxazole	Recov
32	Wound infection, superficial	Ukn	*Ax*/No	Ukn	Cefepime	Recov
33	Meningitis, deep	15	*Ax*/No	Gunshot	Cotrimoxazole/Ceftazidime	Recov
34	Liver abscess, deep	150	*Ax*/No	Ukn	Ukn	Recov
35	Liver abscess, deep	1140	*Ax*/No	Ukn	Ukn	Death
36	Liver abscess, deep	660	*Ax*/No	Ukn	Ukn	Death
37	Perinephric abscess, deep	730	Ax/No	Ukn	Levofloxacin/ Cotrimoxazole, Cefoperazone/Sulbactam, Levofloxacin/Cotrimoxazole	Recov
38	Liver abscess, deep	16	*Ax*/*Escherichia coli*	Ukn	Cefpirome, Colistin	Recov
39	Wound infection, superficial	75	*Ax subsp. Denitrificans Escherichia coli*, *Morganella morganii*	Ukn	Myambutol/ Ciprofloxacin/Clarithromycin	Recov
40	Abscess, deep	3650	*Ax*/*S.epidermidis*, *S. salivarius Mycobacterium avium*	Spread via the auditory canal	Ertapenem, Cefriaxone/ Cotrimoxazole Meropenem	Recov
41	Urinary tract infection, deep	Ukn	*Ax*/No	Ukn	Ciprofloxacin/ Ceftriaxone/Methenamine hippurate	Recov
42	Abscess, deep	14	*Achromobacter* spp./*Stenotrophomonas Maltophilia,* *Candida albicans*	Ukn	Tazobactam/Amikacin	Death
43	Mediastinitis, deep	9	*Achromobacter* spp./*E coli*	Ukn	Tazobactam/Amikacin Cefotaxime/Amikacin, Cefotaxime/Fosfomycin	Recov
44	Wound infection, superficial	53	*Achromobacter* spp./No	Ukn	No antibiotic therapy	Recov
45	Endocarditis, deep	2	*Achromobacter* spp./No	Contamination of the heart-lung machine	Chloramphenicol/ Streptomycin/Sulfadiazine	Recov
46	Wound infection, superficial	11	*Ax*/*Acinetobacter baumannii*	Ukn	Piperacillin/Tazobactam Polymixin B	Recov
47	Abscess deep	Ukn	*Achromobacter* spp./*No*	Endogenous via biliary tract	Piperacillin/Tazobactam	Recov
48	Abscess deep	Ukn	*Achromobacter Group Vd biovar 1*/*No*	Ukn	Cefazolin Gentamycin	Death
49	Urosepsis deep	30	*Ax*/No	Ukn	Cefuroxime Meropenem Cotrimoxazole	Recov

*Ax*: *Achromobacter xylosoxidans*.

## Data Availability

The datasets supporting the findings of this article are included within the article.

## Supplementary Materials

The following are available online at https://www.mdpi.com/article/10.3390/microorganisms9122471/s1; Supplementary File S1: Detailed search strategy.
