# Supplementary material for: Achromobacter spp. Surgical Site Infections: A Systematic Review of Case Reports and Case Series
Source: Microorganisms. 2021 Nov 30;9(12):2471. doi: 10.3390/microorganisms9122471 (PMC8704055; doi:10.3390/microorganisms9122471)
Supplement: Supplementary file 1 [file microorganisms-09-02471-s001.zip › microorganisms-1434477-supplementary.pdf]

Supplementary file 1: Detailed search strategy

Source	Algorythms	Total number of records found	Records removed before screening	Records excluded on title and abstracts	Records not retrieved	Records excluded after full-text reading	Records included in the study
MEDLINE database	((achromobacter) OR (alcaligenes xylosoxidans)) AND (healthcare-associated-infection OR nosocomial-infection OR surgery OR surgical-site-infection OR ventriculitis OR meningitis OR bacteraemia OR mediastinitis OR abscess OR peritonitis OR endocarditis OR burns) Filters: English, French, Humans, from 1961 - 2020	240	0	173	9	36	22
Cochrane Library database	"Achromobacter" in All Text OR "Alcaligenes xylosoxidans" in All Text - (Word variations have been searched)	17	0	17	0	0	0
Clinicaltrials.gov registry	"Achromobacter OR alcaligenes xylosoxidans"	8	1	7	0	0	0
Google scholar.fr website (French language)	Achromobacter , chirurgie, infection	132	0	129	0	3	0
Google scholar.com website (English language)	Achromobacter, surgery, infection	383	159	204	0	12	10
Theses.fr website	Achromobacter, chirurgie, infection	21	10	9	0	2	0
CPIAS-ile-de-France.fr website	Achromobacter	1	0	0	0	0	1
esin.santepublique france.fr, website	Achromobacter	10	0	9	1	0	0
Manual citation searching	-	8	0	0	0	4	4

Supplementary file 1: Detailed search strategy
